# Serious Video Games: Angels or Demons in Patients With Attention-Deficit Hyperactivity Disorder? A Quasi-Systematic Review

**DOI:** 10.3389/fpsyt.2022.798480

**Published:** 2022-04-27

**Authors:** María Rodrigo-Yanguas, Carlos González-Tardón, Marcos Bella-Fernández, Hilario Blasco-Fontecilla

**Affiliations:** ^1^Servicio de Psiquiatría, Puerta de Hierro Health Research Institute-Segovia de Arana (IDIPHISA)-Hospital Universitario Puerta de Hierro-Majadahonda, Madrid, Spain; ^2^Faculty of Medicine, Universidad Autónoma de Madrid, Madrid, Spain; ^3^Consulting Asistencial Sociosanitario SL, Madrid, Spain; ^4^Department of Psychology, Universidad Pontificia de Comillas, Madrid, Spain; ^5^Centro de Investigación Biomédica en Red de Salud Mental, Madrid, Spain

**Keywords:** ADHD, videogames, cognitive rehabilitation, addiction, revision

## Abstract

**Objective:**

To carry out a quasi-systematic review of the use of serious video games for health as a cognitive rehabilitative tool in patients diagnosed with attention-deficit hyperactivity disorder.

**Method:**

A quasi-systematic review of serious video games used as an evaluative and rehabilitative tool in patients with ADHD was conducted. It included behavioral patterns in the use of video games and addiction problems in this population. For its elaboration the PRISMA GUIDES were followed. The search was carried out in three PubMed databases, MEDLINE, and PsycInfo using the keywords: [game OR serious game OR computer game) AND (psychotherapy OR rehabilitation OR intervention OR mental disorders) AND (adhd)], [(adhd) AND (Video game addiction)]. All articles written in English, Spanish, or Portuguese from January 1970 to June 2021 were included: those in which reference was made to the use of video games and/or new technologies as a therapeutic and evaluative tool in children and adults diagnosed with ADHD, as well as those that referred to behavioral and clinical patterns in the use of video games.

**Results:**

We found 605 articles of which 128 were reviewed (44 observational studies, 26 quasi-experimental studies, 26 experimental studies, 8 systematic reviews, 9 narrative texts, 6 case reports, 7 pilot studies, 8 systematic reviews, and 2 meta-analyses). Serious video games can be used to ameliorate ADHD symptoms while improving adherence to treatment. Some serious video games show high accuracy properties assessing ADHD features.

**Conclusion:**

Serious video games for health are increasingly being used as a cognitive rehabilitation tool in patients with attention-deficit hyperactivity disorder (ADHD).

**Systematic Review Registration:**

[www.crd.york.ac.uk/prospero], identifier [CRD42021247784].

## Introduction

The use of video games, particularly by children, is increasingly a matter of concern worldwide. Indeed, “Internet gaming disorder” (IGD) is considered a behavioral addiction (1). IGD is associated with male gender (2) and attention-deficit hyperactivity disorder (ADHD) (3), the most prevalent neurodevelopmental disorder worldwide [prevalence ranges between 5 and 10%; (4)]. Despite the problematic side of the relationship between ADHD and video games, some video games, particularly those called “serious” video games, might be useful in either the diagnosis or treatment of ADHD. Serious video games are defined as those video games specifically designed for educational or health purposes, in contrast with “non-serious,” regular or commercial video games, whose purpose is mostly entertainment. Design of serious video games shares common characteristics with commercial video games. Thus, advances in commercial video games design (e.g., music and graphics designed to make video games more attractive) are also advantageous for serious video games. At the same time, serious video games also take advantage of developments in cognitive and health sciences, such as learning and reward theories or developments of cognitive tasks for psychological therapy (5). Unfortunately, there are virtually no articles trying to balance the pros and cons of the relationship between video games and ADHD. Accordingly, a balanced and constructive review on this topic needs to be offered.

Children with ADHD are particularly vulnerable to developing a severe addiction to gaming (6). Indeed, we have recently reported that ADHD was associated with a three-fold risk of having IGD, but this association was buffered by good social adjustment (7). Besides this negative side of the ADHD–IGD relationship, the use of some video games can help improve the accuracy of ADHD diagnosis. For instance, we have recently reported that an infinite runner-based computer game assisted a clinician in improving the diagnosis of ADHD (8). Another positive use of video games is as a treatment tool for patients with ADHD. Wilkinson et al. (9) and, more recently, other authors (10–14) published systematic reviews concluding that video games might be used as a therapeutic tool in several mental disorders, including ADHD. Vilani et al. (15) concluded that video games can be used to improve emotional regulation and intelligence, and was called into action to introduce video games at both educational and psychological levels. Indeed, several video games have been created for the cognitive rehabilitation of patients with ADHD. The video game “Braingame Brian” (16) promoted some improvement in children with ADHD, including executive functioning (17). Also, regular practice with the video game “Plan-it Commander” helped improve time management, organization, planning, and prosocial skills (18). Similar results were reported with several video games such as “ENGAGE” (19), “Adventurous Dreaming Highflying Dragon” (20), “Movi-Kids” (21), “SmartBrain” (22),”SmartMind” (23), and Akili Interactive (24). These two positive areas of video game use are summarized by a recent systematic review concluding that video games can be used either to diagnose or treat ADHD (25).

Serious video games may also be used to evaluate cognitive functions related to ADHD. Instances of this are Cyber Cruise (26) and CogMed Working Memory training (27) for memory, and Cuibrain and Boogie Academy (28).

In particular, several video game versions of the CPT are available, such as MOXO-CPT (29), Kinect-based CPT (30), virtual reality-based CPT (31), the virtual classrooms ClinicaVR: Classroom CPT (32), and AULA Nesplora (33), as well as general purpose games such as EndeavorRX (34), Empowered Brain, and The Secret Trail of the Moon (35).

The aim of the present study is to systematically review all sound scientific literature published to date about the relationship between video games and ADHD considering three prisms: (1) the problematic (addictive) use of video games, (2) the potential use of video games to improve the diagnosing of ADHD, and (3) the potential therapeutic role of video games in patients with ADHD. We think that a holistic, comprehensive view of this interesting relationship will assist developers and health staff in the development of serious video games with diagnostic and therapeutic properties, as well as the inclusion of some ethics into the development of such tools (i.e., avoiding the addictive properties of some video games in a particularly vulnerable population, those diagnosed with ADHD).

## Materials and Methods

We have developed a quasi-systematic review following the PRISMA guidelines^1^ with the aim of providing the scientific community with an overview of the potential use of serious video games for health as a therapeutic tool for cognitive rehabilitation in patients diagnosed with ADHD. For this, we have taken into consideration all the scientific literature written about cognitive rehabilitation through video games in ADHD, behavioral patterns during their use, and the growing problem of addiction to new technologies in said subpopulation.

Our systematic review has been prospectively registered in the PROSPERO register of systematic reviews, where it is provisionally published as it was submitted (CRD42021247784).

We proceeded to perform in June 2021 a first search in PubMed, PsycInfo, and Medline databases, in keeping with other recent systematic reviews in the field (5, 36, 37), under the two terms (1) “(game OR serious game OR computer game) AND (psychotherapy OR rehabilitation OR intervention OR mental disorders) AND (adhd)”; and (2) “(adhd) AND (Video game addiction).”

Finally, we conducted a new search with the same terms by using the search engine in Google Scholar^2^ (10 first pages). All those articles with scientific rigor published in English, Spanish, or Portuguese were selected from January 1970 to June 2021: those that referred to the use of video games and/or new technologies as a therapeutic and evaluative tool in children and adults diagnosed with ADHD, as well as those that alluded to behavioral and clinical patterns in the use of video games.

Excluded were those in which the game was used only as an evaluation method without providing further relevant data, those lacking scientific rigor, and all those in which there was no explicit reference to the existence of a relationship between the use of video games and clinical patterns of ADHD.

In total, 605 articles were selected, of which 339 were eliminated because they were not related to the purpose of our study and 70 because they were repeated. Of the remaining 196, 4 were discarded because they were in a language in which none of the authors were competent, 9 were eliminated for not focusing on the relationship between ADHD and video games, 47 were eliminated for not providing knowledge relevant to the subject of study, in 5 we could not access the full article because of lack of library funds, and 6 were discarded for other reasons. Finally, to the 125 articles reviewed, 3 new articles found in the reading of other articles were added.

**FIGURE 1:**
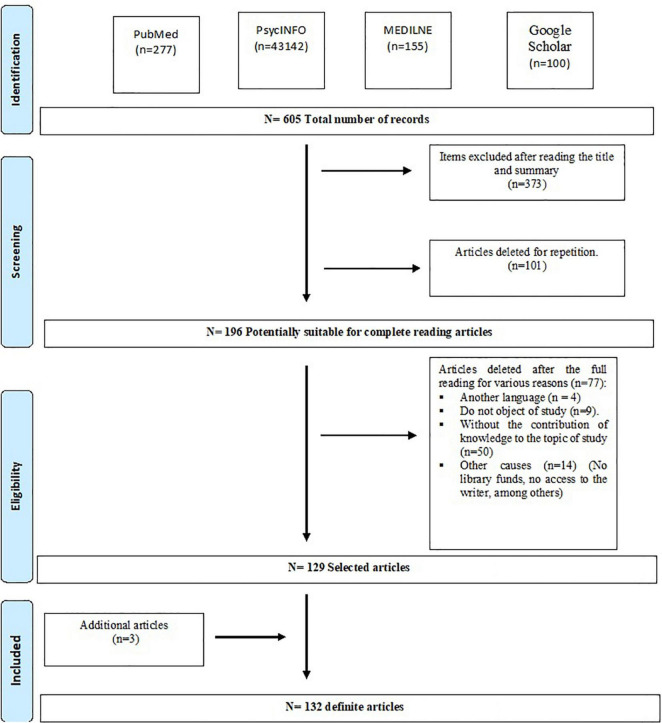
Flowchart of the review following the PRISMA guidelines.

A total of 128 articles were reviewed, of which 44 were observational studies, 26 were quasi-experimental studies, 26 were experimental studies, 9 were narrative texts, 8 were systematic reviews, 7 were pilot studies, 6 were case reports, and 2 were meta-analyses. Figure 1 shows the diagram flow with the decission process.

All studies were classified according to their level of evidence for primary research questions,^3^ ranging from level I (high quality randomized trial or prospective study) to level V (expert opinion).

## Results

In order to address the main theme of our research from a general framework that encompasses the beneficial study of the use of video games in patients with ADHD, but also behavioral and clinical patterns that may become maladaptive in their use, we decided to distribute the 128 articles among three tables: the use of video games as a cognitive rehabilitation tool (*n* = 49), video games as an evaluation tool (*n* = 11), and behavioral and clinical patterns in the use of video games (*n* = 23).

Table 1 refers to the use of video games as a tool aimed at treating ADHD. Serious video games for health can produce significant improvements in attention (17, 22, 28, 38–43), hyperactivity and impulsivity (17, 19, 20, 23, 38, 40, 44–46), executive functions (16, 18, 24, 41, 45, 47), memory (39, 41, 47–54), reading-writing skills (11, 41, 43, 55, 56), emotional regulation (19, 36, 37, 52, 55, 57), motor skills (18, 23, 28), and visual skills (53, 58, 59), among other advantages (18, 47, 48, 51, 55). These improvements may in turn have a beneficial effect in school performance (21, 50).

**TABLE 1:** Serious video games for health as a therapeutic tool in ADHD patients.

Author	Title	Method	Results	Conclusion
**Level I: High quality randomized trial or prospective study; testing of previously developed diagnostic criteria on consecutive patients; sensible costs and alternatives; values obtained from many studies with multiway sensitivity analyses; systematic review of Level I RCTs and Level I studies.**				
Larose et al. (59)	Psychology of computers: XIV. Cognitive rehabilitation through computer games	C = Canada. *N* = 60 (8–14 years; n = 26 children had minimal brain damage; *n* = 24 had attention problems without brain damage). Two groups: trainer with Super Breakout game (n = 40) vs. control group (n = 20). M/F = 50 males/10 females. T = Experimental study with control group Task: Intervention 12 h per week during 1 month	Experimental group improve in visual scan (*p* < 0.05) and visual follow-up (*p* < 0.05)	The serious videogame can be used like tool of visuo-spatial cognitive training
Kaduson and Finnery (57)	Self-control game interventions for attention-deficit hyperactivity disorder.	C = United States. *N* = 63 children with ADHD (8–12 years). Three groups: control group (*n* = 21) vs. “The Self-Control Game” (SCG) (*n* = 21) vs. “Biofeedback game” (*n* = 21). M/F = 58 males/5 females. T = Experimental study with control group. Task: Intervention 10 sessions (1 h each one) during 11 weeks	Outcomes of self-control self-reports of children who received SCG and Biofeedback training was higher [*F*(2, 59) = 4.23, *p* = 0.02] (*p* < 0.01), but their parents self-reports [*F* (2.59) = 0.84, *p* = 0.44] and their behavioral records did not find significant improvements [*F* (2.59) = 69, *p* = 0.50]	Self-control training can reduce self-perception of disinhibition
Shaffer et al. (55)	Effect of Interactive Metronome^®^ Training on Children With ADHD	C = United States. *N* = 56 children with ADHD (6–12 years). Three groups: Metronome training (*n* = 19) vs. Videogame training (*n* = 19) vs. control group (*n* = 18). M/F = 56 males. T = Experimental study with control group. Task: Training during 15 h	Metronome training group improved in five parameters: attentional motor control, language processing, reading, regulation of emotions, and aggressive behavior (*p* ≤ 0.0001)	Children with ADHD who receive metronome training seem to be able to improve in their ADHD symptoms like attention, motor control, and school skills
Shalev et al. (43)	Computerized Progressive Attentional Training (CPAT) Program: Effective Direct Intervention for Children with ADHD	C = Israel. *N* = 36 children with ADHD (6–13 years). Two groups: CPAT training (*n* = 20) vs. control group (n = 16). M/F = 30 males/6 females. T = Experimental study with control group. Task: Training during 1 h, 2 times per week during 2 months	Children with ADHD who received training with CPAT improved in reading comprehension (*p* > 0.05), copy task (*p* > 0.05), and they had reduction of inattention symptoms (*p* > 0.01)	CPAT training program can enhance academic performance and inattention symptomatology
Prins et al. (52)	Does Computerized Working Memory Training with Game Elements Enhance Motivation and Training Efficacy in Children with ADHD?	C = Netherlands. *N* = 52 children with ADHD (7–12 years). Two groups: Control group (*n* = 25) vs. training with videogame (*n* = 27). M/F = 42 males/10 females. T = Experimental study with control group Task: Trainer with a videogame	Generally, Training group had better output in the task (less wrongs), best performance in memory task (*t* = 3.075, df = 26, *p* < 0.01) and their encouragement was higher (*p* < 0.01)	Video games can be use like trainer in memory with ADHD patients
Dovis et al. (47)	Improving Executive Functioning in Children with ADHD: Training Multiple Executive Functions within the Context of a Computer Game. A Randomized Double-Blind Placebo Controlled Trial.	C = Netherlands. *N* = 89 children with ADHD (8–12 years). Three groups: Control group (*n* = 30) vs. Full training group (*n* = 31) vs. partially training group (*n* = 28). M/F = 71 males/18 females. T = Experimental study with control group. Task: 25 sessions training with “Braingame Brain” during 3 months	Children who trained with the videogame improved significantly in short-term memory and visuo-spatial tasks (*p* < 0.001), inhibition (*p* < 0.001), and cognitive-flexibility (*p* < 0.001)	Training with executive-functioning video games can reduce ADHD symptomatology
Sánchez-López et al. (21)	Physical activity intervention (Movi-Kids) on improving academic achievement and adiposity in preschoolers with or without attention deficit hyperactivity disorder: study protocol for a randomized controlled trial	C = Spain. *N* = 1,600 (4–7 years old) from 21 schools. Two groups: Control group (*n* = 697) vs. “Movi-kids” group (n = 891). T = Experimental study. Task: 3 sessions per week (each 60 min) during 2 years	Movi-Kids group increased exercise, school performance, and maintaining a healthy weight (*p* < 0.05)	Increased physical activity results in improvements in school performance and prevents obesity
Weerdmeester et al. (20)	A Feasibility Study on the Effectiveness of a Full-Body Videogame Intervention for Decreasing Attention Deficit Hyperactivity Disorder Symptoms	C = Netherlands. *N* = 73 children with ADHD. Two groups: Control group (Play “Angry Birds Trilogy” *n* = 36) vs. experimental group (Play “Adventurous Dreaming Highflying Dragon” *n* = 37). M/F = 58 males/15 females. T = Experimental study. Task: Play with videogame during 6 sessions (each 15 min)	According to parents and teacher questionnaire, children who played “Adventurous Dreaming Highflying Dragon” improved significantly in two variables: fine motor skills and self-impulsiveness (*p* < 0.01)	“Adventurous Dreaming Highflying Dragon” is a useful cognitive rehabilitation tool to children with ADHD
Bul et al. (48)	Behavioral Outcome Effects of Serious Gaming as an Adjunct to Treatment for Children with Attention-Deficit/Hyperactivity Disorder: A Randomized Controlled Trial	P = Netherlands and Belgium. *N* = 170 children (8–12 years). Two groups: Group 1 (*n* = 88) vs. Group 2 (*n* = 82). M/F = 137 males/33 females. T = Experimental study. Task: Playing with “Plan-it Commander” 3 times per week (each 65 min). Group 1: X0; Group 2: 0X Grupo 1: X0; Grupo 2: 0X X: Training with “Plan-it Commander” during 10 weeks. 0: Conventional therapy during 10 weeks	According to the parent reports, the children in group one improved in time management skills (*p* = 0.004), responsibility (*p* = 0.04), and working memory (*p* = 0.02).	The practice of the game “Play-it Commander” is beneficial for functional improvement of life in children diagnosed with ADHD
Kermani et al. (50)	Working Memory Training in the Form of Structured Games in Children with Attention Deficit Hyperactivity Disorder	P = Iran. N = 60. Two groups: (1) Training with cognitive videogame (n = 30) vs. (2) Control group (n = 30). M/F = 35 males/25 females (8.5–11.2 years old). T = Experimental study with control group. Task: 60-min workout/2 times a week for 12 weeks	The experimental group significantly improved in the parents’ reports in inattentive symptomatology and hyperactivity (*p* = 0.0) and in mathematics (*p* = 0.0), being maintained during the following six months	Working memory training produces transfers in improving school and behavioral performance in children with ADHD
Smith et al. (54)	A Randomized Controlled Trial of an Integrated Brain, Body, and Social Intervention for Children With ADHD.	C = United States and Chinese *N* = 80 (5–9 years old). Two groups: (1) IBBB intervention (n = 42) vs. (2) Control group (*n* = 38). M/F = 53 males/27 females T = Experimental study. Task: Training 15 weeks with the video game “Integrated Brain, Body, and Social” (IBBS)	Only significant improvements were found in working memory tasks (*p* = 0.05)	Training in various domains does not produce widespread improvements in the symptomatology of ADHD
Bikic et al. (39)	A double-blind randomized pilot trial comparing computerized cognitive exercises to Tetris in adolescents with attention-deficit/hyperactivity disorder	C = Denmark N = 18. Two groups: Scientific Brain Training (SBT) (*n* = 9) vs. Tetris game (*n* = 9). M/F = 14 males/4 females. T = Experimental study with control group. Task: 7 weeks of cognitive training	The group with SBT training improved in sustained attention (*p* = 0.0026), while the Tetris group improved in working memory (*p* = 0.0417)	Training with serious health games can serve as attentional and working memory training
Lau et al. (11)	Serious Games for Mental Health: Are They Accessible, Feasible, and Effective? A Systematic Review and Meta-analysis	C = Canada N = Systematic review (*n* = 10 articles); Meta-analysis (*n* = 9 articles). T = Systematic review and meta-analysis	With a regulated and specific training in serious health video games, a reduction in symptoms has been seen in patients with depression, autism, ADHD, alcoholism, and pathologies that affect cognitive functioning	Serious health games have benefits for different pathology groups and ages
Johnstone et al. (49)	Game-based combined cognitive and neurofeedback training using focus pocus reduces symptom severity in children with diagnosed AD/HD and subclinical AD/HD	C = Australia *N* = 85 (9.42 years old). Four groups: ADHD + control (*n* = 22) vs. ADHD + training (*n* = 22) vs. ADHD + control (*n* = 19) vs. ADHD + training (*n* = 22). M/F = 64 males/21 females. T = Experimental study. Task: Training with serious videogame during 25 sessions	According to parents’ evaluations, patients who had video game training had significant improvements in CONEERS-3 questionnaire scores [attention: (*F*[1,80] = 5.375, *p* = 0.023, partial η^2^ = 0.07); hyperactivity/impulsivity: (*F*[1,80] = 9.571, *p* = 0.003, partial η^2^ = 0.11); and in executive functions: (*F*[1,80] = 12.122, *p* = 0.001, partial η^2^ = 0.14)], and CBCL [attention: (*F*[1,80] = 5.821, *p* = 0.018, partial η^2^ = 0.07); aggression: *F*[1,80] = 5.612, *p* = 0.020, partial η^2^ = 0.07), and in outsourcing problems (*F*[1,80] = 10.127, *p* = 0.002, partial η^2^ = 0.12)]	Efficacy of working memory training, inhibitory control, and neurofeedback in children with ADHD
Bruce et al. (58)	Hazard perception skills of young drivers with Attention Deficit Hyperactivity Disorder (ADHD) can be improved with computer based driver training: An exploratory randomized controlled trial.	C = Australia. *N* = 25 (16–25 years old). Two groups: Immediate intervention (*n* = 12) vs. delayed intervention (*n* = 13). M/F = 18 males/7 females. T = Experimental study. Task: Training with “Drive Smart” during 60 min	The group that received immediate training with the video game “Drive Smart” improved in hazard detection (*p* = 0.023)	Perceptual skills training help to reduce traffic accidents in patients with ADHD
Bul et al. (44)	A serious game for children with ADHD: Who benefits the most?	C = Belgium. *N* = 143. Two groups: training with serious videogame during 20 weeks (*n* = 64) vs. control group (medication + training during 10 weeks) (*n* = 79). M/F = 117 males/26 females. T = Experimental study. Task: Play with “Plan-it Commander” during 20 weeks 1 h/3 times per week	After training, children with lower levels of hyperactivity and higher levels of behavioral problems improved in planning and organizational tasks (*p* < 0.05)	The use of serious health video games as a therapeutic tool for ADHD
Bikic et al. (60)	Attention and executive functions computer training for attention-deficit/hyperactivity disorder (ADHD): results from a randomized, controlled trial	C = Denmark *N* = 70. Two groups: (1) Cognitive intervention with ACTIVATE (*n* = 35); (2) Control group (TAU) (*n* = 35). M/F = 59 males/11 females. T = Pilot study. Task = Training with ACTIVATE program during 8 weeks	No significant improvement was found sustained attention (β = -0.047; CI (-0.247 to 0.153), in parent-completed ADHD-SR scales [β = -0.037; CI (-0.224 to 0.150)]; and by ADHD-RS teachers β = 0.093; CI (-0.107 to 0.294); on the BRIEF scales filled out by parents [β = -0.119; CI (-0.307 to 0.069)] and by teachers [β = 0.136; CI (-0.048 to 0.322]	No significant cognitive improvement has been found after training with the ACTIVATE computer program
García-Redondo et al. (28)	Serious Games and their effect improving attention in students with learning disabilities.	C = Spain N = 44 students with ADHD and SLD (6–16 years old). Two groups: control group (*n* = 20) vs. experimental group (*n* = 24). H/M = 27 males/17 females. T = Experimental study with control group. Task: 28 session (10 min each) with 10 games based on multiple intelligences	The experimental group improved in attention performance in D2 test (visual attention task) (*p* < 0.001)	Video games can be used like good cognitive rehabilitation tool in children with ADHD
Benzing and Schmidt (61)	The effect of exergaming on executive functions in children with ADHD: A randomized clinical trial	C = Switzerland *N* = 51. Two groups: (1) Training with exergaming intervention (*n* = 28); (2) Control group (*n* = 23). M/F = 42 males/9 females. T = Experimental study Task = Training with exergaming intervention group [3 session (30 min) per week during 8 weeks]	The group that had exergaming training had a significant improvement in executive functions [*F*(2, 48) = 4.08,*p* = 0.049, d = 0.58] in motor skills [*F*(2, 48) = 7.69, *p* = 0.008, d = 0.80], and in their general psychopathology [*F*(2, 48) = 5.34, *p* = 0.022, d = 0.68]	Exergaming training can benefit executive functions and motor skills
Dovis et al. (62)	Does executive function capacity moderate the outcome of executive function training in children with ADHD?	C = Netherlands *N* = 61. Two groups: (1) Training with “Braingame Brian” (BGB) (*n* = 31) vs. Control group (*n* = 30). M/F = 50 males/11 females. T = Pilot study Task: Training with BGB during 25 training sessions (30–50 min each session)	After training, there is an improvement but not significant in measures of near transfer (EF performance) and far transfer (ADHD symptoms and EF behavior reported by parents) (*p* values needed to be <0.0013 [0.05/38] to survive, whereas actual *p*-values ranged between 0.017 and 0.046)	Patients with ADHD with deficiencies in executive functions do not seem to benefit from prior executive function training
Rajabi et al. (23)	Effect of combined neurofeedback and game-based cognitive training on the treatment of ADHD: A randomized controlled study	C = Iran *N* = 32 adolescents boys with ADHD. Two groups: (1) neurofeedback group + cognitive training “SmartMind” (*n* = 16) vs. (2) Control group (*n* = 16). T = Experimental study with control group. Task: Cognitive training with Neurofeedback during 30 sessions, three times per week	Patients in the training group had a significant improvement in visual attention [(1, 32) = 9.9, *p* < 0.01], in attention [F(1,32) = 20.35, *p* < 0.001], in hyperactivity/impulsivity [F(1, 32) = 32.60, *p* < 0.01] in inhibitory control [*F*(1, 32) = 4.36, *p* < 0.05] in parent-reported ADHD symptoms [*F*(1, 32) = 26.73, *p* < 0.001]. Also, increased activity was observed in SMR waves [*F*(1, 32) = 5.19, *p* < 0.05]	Neurofeedback Training Along with Use of Cognitive Training Games May Reduce ADHD Symptoms
Hahn-Markowitz et al. (45)	Efficacy of Cognitive-Functional (Cog-Fun) Occupational Therapy Intervention Among Children With ADHD: An RCT	C = Israel N = 107 children. Two groups: Training with Cognitive-Functional (Cog-Fun) (n = 54) vs. Control group (n = 53) M/F = 76 males/31 females. T = Experimental study Task: Training with Cog-Fun during 6 months (60 hr theoretical and practical).	Children who received Cog-Fun training showed significant improvements in parent-completed questionnaires (*p* < 0.05): BRIEF (>0.5 SD), CPRS-R(>0.5 SD), and PedsQL (>0.5 SD)	Training in Cog-Fun Occupational therapy (OT) showed positive effects on parents
**Level II: Lesser quality RCT; prospective comparative study; retrospective study; untreated controls from an RCT; lesser quality prospective study; development of diagnostic criteria on consecutive patients; sensible costs and alternatives; values obtained from limited studies; with multiway sensitivity analyses; systematic review of Level II studies or Level I studies with inconsistent results.**				
Prins et al. (16)	“Braingame Brian”: Toward an Executive Function Training Program with Game Elements for Children with ADHD and Cognitive Control Problems.	C = Netherlands. N = 40 children with ADHD (8–12 years). Two groups: waitlist group (n = 22) vs. Training with “Braingame Brian” (n = 18). T = Pilot study. Task: 25 sessions (40–50 minutes each) with “Braingame Brian.”	Braingame Brian training let to improvements in executive functions.	Braingame Brian videogame can be effective as cognitive rehabilitation tool with ADHD children.
Van der Oord et al. (17)	A Pilot Study of the Efficacy of a Computerized Executive Functioning Remediation Training With Game Elements for Children With ADHD in an Outpatient Setting: Outcome on Parent- and Teacher- Rated Executive Functioning and ADHD Behavior.	C = Netherlands. N = 40 children with ADHD (8–12 years). Two groups: Control group (n = 20) vs. Training with video games (n = 20). M/F = 33 males/7 females. T = Pilot study. Task: Training with “Braingame Brian” videogame during 25 sessions.	Children who receive training in videogame had statistical significance ADHD symptoms’ outcomes in DISC-IV and BRIEF questionnaires regardless of medication (p < 0.05).	Braingame Brian is a serious videogame to cognitive rehabilitation tool to ADHD children.
Strahler et al. (36)	ADHD rehabilitation through video gaming: a systematic review using PRISMA guidelines of the current findings and the associated risk of bias.	C = Brazil. N = 14 studies. T = Systematic Review.	Serious videogame for the health is focused on training in the attention, working memory, and change of behavior	Serious videogame can be a good option of cognitive rehabilitation.
Flynn et al. (24)	Solitary Active Videogame Play Improves Executive Functioning More Than Collaborative Play for Children with Special Needs.	C = United States. N = 36 (7–18 years old) with special needs ADHD (61%), ASD (17%), ADHD/ASD (17%), other (6%). Two groups: playing alone (n = 14) vs. playing in pairs (n = 22). M/F = 24 males/12 females. T = Quasi-experimental study. Task: Play alone or in couple for 20 minutes.	Patients who played alone had a significant improvement in the Stroop test (F(1, 33) = 6.70, p = 0.014) and in the Flank task (F(1, 33) = 5.92, p = 0.021)	Training with video games produces more improvement in executive functions if played alone.
Quian et al. (41)	Brain-computer-interface-based intervention re-normalizes brain functional network topology in children with attention deficit/hyperactivity disorder.	C = Singapore. N = 66. Two groups: Control group (N = 22) vs. Intervention group (N = 44). H/M = 66 males. T = Quasi-experimental study. Task: Training during 8 weeks use BCI-based attention.	After training, children with ADHD inattention had better connectivity in salience/ventral attention network (SVN) (p = 0.019) and they had increased functional connectivity between task-positive networks and subcortical regions (P = 0.05). On the other hand, children with ADHD hyperactivity-impulsive improve in prefrontal regions (p < 0.05).	BCI based attention training can be used like rehabilitation cognitive training in children with ADHD.
Moore et al. (51)	Clinician-delivered cognitive training for children with attention problems: effects on cognition and behavior from the ThinkRx randomized controlled trial.	C = United States. N = 13 (8–14 years old). Children with attention problems. Two groups: Control group (n = 7) vs. Cognitive training group (n = 6). M/F = 8 men/5 females. T = Experimental study with control group. Task: 40 Cognitive training sessions with ThinkRx (60–90 minutes session/3 days per week. Totally was 60 h	Children who were treatment groups improve in five quantitative measures auditory processing, working memory, reasoning and logic, long-term memory, and IQ score (p < 0.05), and three qualitative measures: parents reported to improve in confidence, self-discipline and cooperation.	ThinkRx can be new cognitive training tool to improve the cognitive and behavioral parameters.
Flynn et al. (24)	A Game-Based Repeated Assessment for Cognitive Monitoring: Initial Usability and Adherence Study in a Summer Camp Setting.	C = United States N = 130 children with different pathologies: ADHD (32%), ASD (31%), ADHD/ASD (20%), or other mental disorder (17%). M/F = 76 males/24 females. T = Quasi-experimental study. Task: Play to Akili during 2 weeks at a Summer Camp.	Generally, performance outcomes of activities was better by children with ADHD (p < 0.05): (For example, simple and multi-task reaction time: F(3,54) = 2.51, p = 0.068; t-test, p = 0.066).	Children reported to Akili Interactive’s Monitoring were enjoyable and the rate of adherence was high.
Kokol et al. (37)	Serious Game-based Intervention for Children with Developmental Disabilities	C = Slovenia N = 145 studies. T = Systematic review.	The main pathologies with the highest number of articles were: Autism spectrum disorder (n = 45); Developmental coordination disorder (n = 26); Attention deficit hyperactivity disorder (n = 24); Disabilities affecting intellectual abilities (n = 23).	Serious health games can be potential tools for anxiety reduction, stress regulation, emission recognition, and rehabilitation.
López et al. (64)	The plausibility of using unmanned aerial vehicles as a serious game for dealing with attention deficit-hyperactivity disorder.	C = Mexico N = 13 studies. T = Systematic Review	Study of the possible use of “Brain computer interfaces” in patients with ADHD for the improvement of a serious health game “Ummaned Aerial Vehicles.”	The use of “Brain computer interfaces” seems to be an effective tool for cognitive training in patients with ADHD.
**Level IV: Case series; case control study (diagnostic studies); poor reference standard; analyses with no sensitivity analyses.**				
Lim et al. (40)	A Brain-Computer Interface Based Attention Training Program for Treating Attention Deficit Hyperactivity Disorder.	C = Singapore. N = 20 children with ADHD without medication. M/F = 16 males/4 females. T = Experimental study. Task: 24 sessions during 8 weeks.	Patients who played with the BCI videogame improved in attention symptom, hyperactivity and impulsivity (p < 0.01).	BCI videogame is a new cognitive rehabilitation tool to children with ADHD.
Frutos-Pascual (65)	Adaptive Tele-Therapies Based on Serious Games for Health for People with Time-Management and Organizational Problems: Preliminary Results.	C = Brazil. N = 17 children without ADHD (12–19 years). M/F = 10 males/7 females. T = Descriptive study. Task: 8 weeks training with “Summer Treatment Program-Adolescent” (STP-A).	Children reported being satisfied with the program, especially on prioritizing task (78.75 out of 100).	Videogame can be used to train in planification to children with or without ADHD in their daily life.
Rohani et al. (42)	Brain-Computer Interface using P300 and Virtual Reality: A Gaming Approach for Treating ADHD.	C = Denmark N = 5 healthy children. T = Descriptive study. Task: Playing with VR Videogame two activities: ANISPELL and T-SEARCH.	P300 potential can be used like measure of attention in videogame (Subject 1 had the smallest averaged statistical significance (t(1,9) = 5.78, p < 0.0153) and Subject 4 had achieved the largest (t(1,9) = 17.31, p < 0.0022). To enhance the effect of cognitive rehabilitation it is necessary to use auditive and visual distractors and low-error-rate.	Serious videogame can be used to train in sustained attention.
Shih et al. (46)	Assisting children with Attention Deficit Hyperactivity Disorder to reduce the hyperactive behavior of arbitrary standing in class with a Nintendo Wii Remote Controller through an active reminder and preferred reward stimulation	C = Chinese N = 2 children with ADHD (7–10 years). M/F = 2 boys. T = Case report. Task: 11 training sessions with “Nintendo Wii remote”	Both patients had a significant improvement in self-control of hyperactivity (p < 0.01).	“Nintento Will remote” can be used to self-control therapy with ADHD children.
Wrońska et al. (56)	An iPad-Based Tool for Improving the Skills of Children with Attention Deficit Disorder.	C = Spain N = 6 children with ADHD (8–12 years). M/F = 2 males/4 females. T = Experimental study without control group. Task: Training with LyC videogame.	Task completion average time was decreasing from first task (μ = 86.17 ± 19.53), to second task (μ = 50.33 ± 18.04), and third task (μ = 40.00 ± 15.07).	“LyC” videogame is a good tool to improvement of comprehensive reading and sustained attention in children with ADHD regardless of age.
Bul et al. (18)	Development and User Satisfaction of “Plan-It Commander,” a Serious Game for Children with ADHD.	C = Netherlands N = 42 children with ADHD (8–11 years). T = Pilot study without control group. Task: Training with “Plan-it commander” videogame during 8 week (30–45 each session).	Outcomes parents questionnaires indicated a good satisfaction with the “plan-it commander” videogame: 6,7; SD = 1.4 (on a scale from 1 to 10). 67% Children who played to “Plan-it Commander” videogame reflected to learning in management time, planning/organizing and prosocial skills.	“Plan-It Commander” videogame is a cognitive rehabilitation tool to improve in management time, planning, and prosocial skills.
Ali and Puthusserypady (38)	A 3D Learning Playground for Potential Attention Training in ADHD: A Brain Computer Interface Approach.	C = Denmark. N = 11 healthy subjects (27.5 ± 4.5 years). M/F = 8 males/3 females. T = Experimental study Task: Training with Virtual Reality 3D.	Healthy subjects improved in sustained attention and inhibition of stimulus task (average hits = 92.26 ± 7.97; and average time = 3.07 ± 1.09).	3D serious video games can be used to cognitive rehabilitation in focus attention with healthy children and children with ADHD.
Ruiz-Manrique et al. (53)	Case Report: “ADHD Trainer”: the mobile application that enhances cognitive L in ADHD patients.	C = Spain. T = Case report. Boy with ADHD (10 years old). Task: Training with “ADHD trainer” videogame.	After training period, outcome was better in three questionnaires: Conners Parents Questionnaire (T0 = 20, T1 = 16), Conners Teacher Questionnaire (T0 = 19, T1 = 15) and Barkley School Situations Questionnaire (T0 = 70, T1 = 66).	“ADHD Trainer” videogame improved in visuo-spatial memory, fine motor skills, and decrease to video games addiction.
Healey and Halperin (19)	Enhancing Neurobehavioral Gains with the Aid of Games and Exercise (ENGAGE): Initial open trial of a novel early intervention fostering the development of preschoolers’ self-regulation.	C = Australia. N = 25 (3–4 years old) M/F = 19 males/6 females T = Experimental study. Task: Play to ENGAGE videogame during 5 weeks.	Parents reported in their children a decrease of the hyperactivity (p > 0.001), the aggressiveness (p < 0.001), and attentional problems (p < 0.001). These effects maintained over the following 12 months.	ENGAGE videogame is a therapeutic tool to training in self-regulation with preschool children.
Bégel et al. (67)	Music Games: Potential Application and Considerations for Rhythmic Training.	C = France N = 27 articles. T = Narrative review.	As of the present day, there is no rhythmic skills training video game that meets the essential requirements to serve as a rehabilitation tool.	Video games based on rhythmic skills training can be useful in psychiatric pathologies such as ADHD.
Buitelaar (63)	Optimising treatment strategies for ADHD in adolescence to minimise ‘lost in transition’ to adulthood.	C = Netherlands T = Narrative review.	Use of mobile applications and mindfulness to improve medication adherence in adolescents with ADHD.	Use of alternative methods to improve medication adherence in adolescents with ADHD.
Savulich et al. (68)	Focusing the Neuroscience and Societal Implications of Cognitive Enhancers.	C = United Kingdom T = Narrative review.	Cognitive training based on serious health video games can improve motivational, attentional, and reasoning symptoms.	Importance of multimodal treatment (medication and cognitive training) in patients with prefrontal lobe involvement
Rivera-Flores & Vera-Álvarez (22)	Intervención computarizada para mejorar la atención sostenida en un niño con TDAH.	C = Peru T = Case report (9 years old) Task = Training with “Smartbrain” game during 16 sessions (1 h per session)	The patient had an improvement in focus attention as assessed by the CSAT program after the intervention (d’ (T pre-test: 0 = 0; T post-test: 39); A’ (T pre-test: 15; T post-test: 37).	Computer-based attention training can be useful as a cognitive tool.
Bossenbroek et al. (64)	Efficacy of a Virtual Reality Biofeedback Game (DEEP) to reduce anxiety and disruptive classroom behavior: Single-Case study.	C = Netherlands N = 8 adolescents attending a secondary special school (n = 5 with ADHD). M/F = 7 males/1 female. T = Pilot study with design ABAB (A = No intervention; B = Intervention). Task: Intervention with DEEP 6 session during 4 weeks.	After training there was a small significant reduction in anxiety (d = -0.29) and disruptive behavior although this did not reach significance (d = -0.16). The state of relaxation lasted an average of 2 h	Virtual Reality as a potential anxiety reduction tool.
**Level V: Expert opinion**				
Walker et al. (69)	Play Attention Interactive Learning Tool.	C = United States. T = Narrative Text.	To achieve a therapeutic effect is necessary to continued practice at least during 40 h (or if existing behavior problems, is necessary to increase in 60 h) with “play attention” videogame.	It is necessary carry out more research about the effect therapeutic with “Play attention” program.
Wilkinson et al. (9)	Online Video Game Therapy for Mental Health Concerns: A Review.	C = Canada. T = Narrative text.	Recently, serious video games have been designed like rehabilitation cognitive tool for different pathologies: violent behaviors, anxiety disorders, ADHD, autism, and neurodegenerative disorders.	Serious video games are used in mental health.
Benzing et al. (70)	Cognitively and physically demanding exergaming to improve executive functions of children with attention deficit hyperactivity disorder: a randomized clinical trial.	C = Swiss. N = 66 children with ADHD (8–12 years old). T = Narrative text about future research.	An experimental study will be done with control group (n = 66; 8–12 years). The training will be with video games that mix the cognitive and motor component. The training will be during 3 sessions per week (each 30 min) during 2 months.	Importance of cognitive training and sports practice in children with ADHD.
Goodwin (71)	Attention training for infants at familial risk of ADHD (INTERSTAARS): study protocol for a randomized controlled trial.	C = England. N = 50 children. (10–14 months). Two groups: control group (n = 25) vs. “INTERSTAARS” group (n = 25). T = Narrative text about future research. Task: Attentional training with INTERSTAARS videogame during 9 weeks.	An experimental care training study will be done with the “INTERSTAARS” program in children aged 10–14 months at risk of developing ADHD.	INTERSTAARS is the first cognitive training instrument in babies at risk of developing ADHD.

*ADHD, attention deficit hyperactivity disorder; SMR, sensory motor; SLD, specific learning disorder.*

Nonetheless, some studies (54, 60, 61) did not find evidence of any improvement in ADHD symptomatology. Rajabi et al. (23) found improvement in impulsivity, but not in attention. Bul et al. (44) showed greater improvement in hyperactivity and behavioral problems in moderate patients compared with patients with more severe symptoms.

Serious video games may also improve adherence to treatment (24, 63). This positive effect may be related to patients perceiving video games as enjoyable activities (24, 65), apart from the perception of improvement in the patient’s quality of life (45, 48) and anxiety (37, 66), among others.

Table 2 shows the articles that refer to the use of video games as an evaluation tool for the diagnosis of ADHD. Different video games have been designed to evaluate the presence and severity of ADHD nuclear symptoms (30, 72–77), prospective memory (26), executive functions (78), stress (79), and prosocial behavior (80). Serious video games showed high sensitivity and specificity values (73, 75, 76), as well as a high correlation with CPT measurements (8, 72) and measurements similar to neuropsychological tests (73).

**TABLE 2:** Serious video game for health as an assessment tool in ADHD patients.

**Level II: Lesser quality RCT; prospective comparative study; retrospective study; untreated controls from an RCT; lesser quality prospective study; development of diagnostic criteria on consecutive patients; sensible costs and alternatives; values obtained from limited studies; with multiway sensitivity analyses; systematic review of Level II studies or Level I studies with inconsistent results.**				
Mitchell et al. (74)	Reaction Time, Impulsivity, and Attention in Hyperactive Children and Controls: A Video Game Technique	C = United States. *N* = 201 (5–13 years old). Two groups: ADHD group (*n* = 49) vs. Control group (*n* = 152). M/F = 115 males/86 females T = Quasi-experimental study. Task: to evaluate the new video game	The objective has been the creation of an evaluation video game. Children with ADHD have a more variable performance, slower and make more mistakes (*p* < 0.0001)	Children with ADHD have lower motor and calculating speeds
Kerns and Price (26)	An Investigation of Prospective Memory in Children With ADHD.	C = Canada. T = Two quasi-experimental studies: Study 1: (*n* = 20, 8–13 years old). Two groups: ADHD (*n* = 10) vs. Control (*n* = 10). Study 2: (*n* = 42, 6–13 years old). Two groups: ADHD (*n* = 21) vs. Control (*n* = 21)	Children with ADHD performed worse in the CyberCruiser game in both Experiment 1 (*p* < 0.001) and Experiment 2 (*p* < 0.01)	The CyberCruiser video game as a way to evaluate prospective memory
Lawrence et al. (78)	Executive function and ADHD: A comparison of children’s performance during neuropsychological testing and real-world activities	C = Australia *N* = 44 males (6–12 years old). Two groups: ADHD group (*n* = 22) vs. Control group (*n* = 22). T = Quasi-experimental study. Task: Evaluation with neuropsychological tests and two video games	Children with ADHD performed less well in executive functions and processing speed in both neuropsychological tasks and real-world activities (*p* < 0.05)	Children with ADHD have problems with executive functions
Ohan and Johnston (80)	What is the Social Impact of ADHD in Girls? A Multi-Method Assessment	C = Canada. *N* = 80 females (9–12 years old). Three groups: ADHD + ODD (*n* = 22) vs. TDAH (*n* = 18) vs. Control group (*n* = 40). T = Quasi-experimental study. Task: Play with video games “Girl’s club”	Girls with ADHD and ODD showed less prosocial and more aggressive behaviors (*p* < 0.001)	The Girls Club video game as a method of assessing prosocial behavior
Pop-Jordanova and Gucev (79)	Game-based peripheral biofeedback for stress assessment in children.	P = Republic of Macedonia *N* = 120 (9.33 ± 1.63 years old). Four groups Cystic Fibrosis (*n* = 30) vs. Generalized anxiety (*n* = 30) vs. ADHD (*n* = 30) vs. control group (*n* = 30). M/F = 60 males/60 females. T = Quasi-experimental study. Task = Use of biofeedback as an evaluation method	In children with generalized anxiety and ADHD more psychopathological features were found, less extroversion (*p* < 0.001) and more omissions due to lack of attention (*p* < 0.05). Lower scores on lying scales were found in children with ADHD (*p* < 0.01)	Relaxation is a more difficult task for children with ADHD and generalized anxiety
Heller et al. (73)	A Machine Learning-Based Analysis of Game Data for Attention Deficit Hyperactivity Disorder Assessment.	C = United States. *N* = 52. Two groups: ADHD (*n* = 26) vs. No ADHD (*n* = 26). M/F = 26 males/26 females. T = Quasi-experimental study. Task: Testing the effectiveness of a new diagnostic tool for ADHD: “Groundskeeper”	Diagnosis efficacy for ADHD was high for inattention type 78% (*p* > 0.05), ADHD, combined type 75% (*p* < 0.05); anxiety disorders, 71% and depressive disorder 76%	The “’Groundskeeper” game seems to be useful for the diagnosis of ADHD
Berger and Goldzweig. (29)	Response Inhibition in Preschoolers at Familial Risk for Attention Deficit Hyperactivity Disorder: A Behavioral and Electrophysiological Stop-Signal Study.	P = Israel. N = 60 males (5 years old). H/M = 60 hombres. T = Quasi-experimental study. Task = play the computer game “stop-signal”	Children with increased symptoms of ADHD, especially with parent-reported hyperactivity (*p* < 0.05) had less activity in the regions controlling inhibition	Inhibitory behavior can be a predictor of ADHD
Peijnenborgh et al. (76)	A Study on the Validity of a Computer-Based Game to Assess Cognitive Processes, Reward Mechanisms, and Time Perception in Children Aged 4–8 years	C = Netherlands *N* = 136 (4–8 years old). Two groups: ADHD (*n* = 40) vs. non-pathological (*n* = 96). M/F = 73 males/63 females. T = Quasi-experimental study. Task: Play the video game Timo’s adventure	Older children made fewer inhibition errors (*p* = 0.001), had faster reaction times (*p* < 0.001) and more accurate interval times (*p* < 0.001)	Timo’s Adventure obtained high sensitivity (0.89) and specificity (0.69) for detecting children with ADHD
Faraone et al. (72)	The Groundskeeper Gaming Platform as a Diagnostic Tool for Attention-Deficit/Hyperactivity Disorder: Sensitivity, Specificity, and Relation to Other Measures.	C = United States. *N* = 113 (6–17 years old) Two groups: ADHD (*n* = 66) vs. Non-ADHD (*n* = 47). T = Quasi-experimental study. Task: Assessment with CPT-3 and “the groundskeeper gaming platform”	A significant correlation was found between the CPT-3 assessment test and the groundskeeper gaming platform (*p* < 0.01)	The use of video games the groundskeeper gaming platform” as an evaluation tool of the adhd
Delgado-Gomez et al. (30)	Microsoft Kinect-based Continuous Performance Test: An Objective Attention Deficit Hyperactivity Disorder Assessment.	C = Spain *N* = 30 (8–12 years old) M/F = 21 males/9 females T = Quasi-experimental study Task: New assessment tool based on the CPT	The video game obtained a significant correlation with the symptoms of inattention (*p* = -0.11), hyperactivity (*p* = -0.29), and impulsivity (*p* = -0.37) finding a relationship between commission detection and inattention (*p* = -0.03), hyperactivity (*p* = 0.01), and impulsivity (*p* = 0.24)	“Microsoft Kinect-based Continuous Performance Test” as an improvement of the CPT for the assessment of ADHD
Mwamba et al. (75)	PANDAS: Paediatric Attention-Deficit/Hyperactivity Disorder Application Software	C = South Africa *N* = 30 children (Non-ADHD (*n* = 19); ADHD (*n* = 11). M/F = 16 males/14 females. T = Quasi-experimental study. Task = Performing the LOOCV task	The detection sensitivity of the diagnosis of ADHD with the LOOCV task was 75%	Further research is needed into the use of video games as a diagnostic tool

*ODD, oppositional defiant disorder.*

Table 3 groups articles related to various behavioral patterns in patients with ADHD when they are using video games. Patients with ADHD show greater problems in attention (81–83), motor control tasks, and working memory tasks (84). They also have difficulty inhibiting responses and make more risky decisions (85, 86), their performance decreases when reinforcement is delayed, and they seem to be more sensitive to reward (87) and punishment (88). Likewise, several studies concluded that a relationship exists between ADHD and intrinsic factors such as male gender and greater number of hours a day in the use of video games; along this line, several studies found that playing more than 1 h per day may worsen ADHD symptoms (82, 83).

**TABLE 3:** ADHD behavioral patterns with video game use.

**Level II: Lesser quality RCT; prospective comparative study; retrospective study; untreated controls from an RCT; lesser quality prospective study; development of diagnostic criteria on consecutive patients; sensible costs and alternatives; values obtained from limited studies; with multiway sensitivity analyses; systematic review of Level II studies or Level I studies with inconsistent results.**				
Lawrence et al. (84)	ADHD Outside the Laboratory: Boys’ Executive Function Performance on Tasks in Videogame Play and on a Visit to the Zoo	C = Australia. N = 114 males (9.6 ± 2.1). Two groups: ADHD (*n* = 57) vs. Control group (*n* = 57). T = Quasi-experimental study. Task: Play 3 video games: “Point Blanck,” “Crash Bandicoot,” and “Zoo.”	The ADHD group in the video game “crash Bandicoot” in tasks with higher work memory load spent more time to complete it (*p* < 0.01) and performed more self-tests (*p* < 0.001); in the video game “zoo” they had more problems of behavioral inhibition (*p* < 0.01) and motor control (*p* < 0.01)	Cognitive difficulties derived from ADHD are context-dependent and are associated with some behavioral inhibition deficits
Shaw et al. (102)	Inhibition, ADHD, and Computer Games: The Inhibitory Performance of Children with ADHD on Computerized Tasks and Games.	C = United Kingdom *N* = 32 (6–14 years old). Two groups: ADHD (*n* = 16) vs. control group (*n* = 16). M/F = 29 males/3 females T = Quasi-experimental study. Task: Study the relationship between inhibitory control and video games	Children with ADHD showed difficulty in inhibiting classic assessment tasks such as CPT-II compared to the video game Pokémon Task [*F*(1,15) = 15.67, *p* = 0.05]	Children with ADHD have better impulse control in engaging video games
Aase and Sagvolden (81)	Infrequent, but not frequent, reinforcers produce more variable responding and deficient sustained attention in young children with attention-deficit/hyperactivity disorder (ADHD)	C = Norway. *N* = 56 males (6–12 years old). Two groups: ADHD (*n* = 28) vs. Control group (*n* = 28). T = Quasi-experimental study. Task: play a computer game	Children with ADHD had problems in sustained attention and their performance was worse (*p* < 0.01) when reinforcement was infrequent	Children with ADHD have an altered reinforcement mechanism
Aase and Sagvolden (81)	Decision-making on an explicit risk-taking task in preadolescents with attention-deficit/hyperactivity disorder.	C = Switzerland *N* = 47 (11–13 years old). Two groups: ADHD (*n* = 23) vs. Control group (*n* = 24). T = Quasi-experimental study. Task: Play the video game “Game of Dice Task”	A moderate relationship between hyperactivity and risky decisions was observed in the first game (*p* < 0.05), increasing this relationship in the second game (*p* < 0.001)	There is an association between risky decision making and impulsiveness
Tahiroglu et al. (83)	Short-Term Effects of Playing Computer Games on Attention	C = Turkey *N* = 101 (9–12 years old). Two groups: Psychiatric disorder (*n* = 82) vs. Non-psychiatric disorder (*n* = 19). M/F = 64 males/37 females. T = Quasi-experimental study. Task: play a computer game on attention	Being male (*p* > 0.048), young (*p* > 0.044), number of hours of video game playing (>1 h/day *p* > 0.015; > 1 h/day *p* < 0.013) and symptoms of inattention in ADHD (*p* < 0.050) may have an effect on the Stroop test	The time spent playing video games can have an effect on short-term attention as measured by the Stroop test
Silva and Frère (89)	Virtual environment to quantify the influence of color stimuli on the performance of tasks requiring attention	C = Brazil. *N* = 40 (15–25 years old). Two groups: ADHD without medication (*n* = 20) vs. Control group (*n* = 20). M/F = 17 males/23 females. T = Quasi-experimental study. Task: Playing the video game “Raiders of the Lost Treasure”	The use of yellow-blue colors decreased performance in both groups (*p* < 0.05) and especially in children with ADHD in attention tasks (*p* > 0.05)	The performance in the daily tasks of people with ADHD is influenced by the color of the same
Dovis et al. (90)	Can Motivation Normalize Working Memory and Task Persistence in Children with Attention-Deficit/Hyperactivity Disorder? The Effects of Money and Computer-Gaming.	C = Belgium. *N* = 61 (9–12 years old). Two groups: ADHD (*n* = 30) vs. control group (*n* = 31). M/F = 41 males/20 females. T = Quasi-experimental study. Four conditions: Feedback vs. 1 euro vs. 10 euro vs. computer game. Task: Study of motivation in patients with ADHD	Children with ADHD needed extra strong reinforcement (incentive 10 euros or video game) (*p* < 0.001)	Video games can be a cost-effective way to maximize performance in children with ADHD
Bioulac et al. (6)	Video Game Performances Are Preserved in ADHD Children Compared With Controls	C = France. *N* = 42 males. Two groups: ADHD (TDAH (*n* = 26, 8.3 ± 0.9 years old) vs. control (*n* = 16, 7.8 ± 0.8 years old). T = Quasi-experimental study. Task: Evaluation with CPT and video game “EyeToy”	The ADHD group performed worse on the CPT-II task (*p* > 0.001) but there was no difference in performance with the video game “EyeToy” (*p* > 0.05)	Difficulties in the inhibitory control of patients with ADHD depend on the task at hand
Lis et al. (86)	Social Interaction Behavior in ADHD in Adults in a Virtual Trust Game.	C = Germany. *N* = 40 (36 ± 10 years old). Two groups: ADHD (*n* = 20) vs. control group (*n* = 20). M/F = 22 males/18 females. T = Quasi-experimental study. Task: Play the game “Trust Game”	The ADHD group made greater investments (*p* = 0.021) regardless of the scenario. Both groups transferred more monetary units in the face of happy stimuli (*p* < 0.001)	Adults with ADHD appear to show alterations in social interaction behavior
Michel et al. (91)	The effect of reinforcement variables on inhibition in children with ADHD	C = Canada. *N* = 40 (7–12 years old). Two groups: ADHD (*n* = 20) vs. control group (*n* = 20). M/F = 26 males/13 females. T = Quasi-experimental study. Task: Play “Fire Fighting Game” with three types of reinforcement: no reinforcement, immediate, and delayed	Children with ADHD performed better when they had shorter delayed reinforcement (*p* < 0.05)	Children with ADHD have greater difficulty with inhibitory control
Robaey et al. (87)	Stop and look! Evidence for a bias toward virtual navigation response strategies in children with ADHD symptoms	C = Canada. N = 256 (8,43 ± 0.11 años). M/F = 115 males/108 females. T = Quasi-experimental study. Task: Playing video games: Unreal; Epic Games, Raleigh, NC	Children with at least one symptom of ADHD performed better in response (*p* = 0.024), but not in learning the task (*p* = 0.038)	Learning based on repetition and reward is more effective in children with ADHD
Bolic et al. (92)	Internet Activities During Leisure: A Comparison Between Adolescents With ADHD and Adolescents From the general Population.	C = Sweden. N = 779 (12–18 years old). Two groups: ADHD (*n* = 102) vs. control group (*n* = 677). M/F = 427 males/388 females. T = Descriptive observational study. Task: Leisure research of teenagers with and without adhd	Teenagers with ADHD went out less with friends, did less sport (*p* > 0.001), read and danced less (*p* < 0.05); However, they played more online games and visited more websites and pornography (*p* > 0.05) and chatted less and did less homework with the Internet (*p* < 0.05)	Teenagers with ADHD prefer to play internet games online
Furukawa et al. (88)	Evidence for increased behavioral control by punishment in children with attention-deficit hyperactivity disorder.	C = New Zealand. *N* = 210 (5–13 years old). Two groups: ADHD (*n* = 145) vs. Control group (*n* = 65). M/F = 146 males/64 females. T = Quasi-experimental study. Task: Playing two video games with different punishment rates	Children with ADHD performed worse when the punishment was prolonged in time (*p* < 0.05)	Children with ADHD are more sensitive to the cumulative effect of punishment
**Level IV: Case series; case control study (diagnostic studies); poor reference standard; analyses with no sensitivity analyses.**				
Chan and Rabinowitz (82)	A cross-sectional analysis of video games and attention deficit hyperactivity disorder symptoms in adolescents	C = United States. *N* = 72 (15.3 ± 0.7 years old). M/F = 31 males/41 females. T = descriptive observational study Task: Study the relationship between ADHD and video game use	A correlation was found between playing more than 1 h a day on the YIAS scale and higher symptoms on the CPRS scale of inattention (*p* < 0.001) and ADHD (*p* < 0.020)	Playing video games more than an hour a day can make ADHD symptoms worse
Swing et al. (93)	Television and Video Game Exposure and the Development of Attention Problems	C = Canada. *N* = 1533 (9.6 years old). Two groups: Middle childhood (*n* = 1,323) vs. early adulthood (*n* = 210). M/F = 706 males/827 females T = Descriptive observational study. Task: Study the relationship between the use of new technologies and attention symptoms	In both groups, exposure to television and video console shows a small moderate correlation with attention problems (r between 0.17 and 0.23)	Watching TV and playing video games can be a risk factor for attention problems
Pfeifer et al. (94)	Play preference of children with ADHD and typically developing children in Brazil: A pilot study	C = Brazil. *N* = 32 (7–12 years old). Two groups: ADHD (*n* = 16) vs. Control group (*n* = 16). M/F = 28 males/4 females. T = Descriptive observational study. Task: Investigate the preference for play in children with ADHD	Children with ADHD preferred to play at school (*p* < 0.05) while the control group preferred on the street (*p* < 0.01) and showed a preference for board games (memory and dominoes) (*p* = 0.01)	Children with ADHD preferred to play in protected places with adult supervision, individual games and no implicit rules
Ferguson (95)	The influence of television and video game use on attention and school problems: A multivariate analysis with other risk factors controlled.	C = United States. *N* = 603 (10–14 years old). M/F = 309 males/294 females. T = Descriptive observational study. Task: To research the influence of new technologies on patients with ADHD	A correlation appears between ADHD and intrinsic factors: being male, antisocial traits, negative interactions with adults and anxiety (*p* > 0.05). The use of video games and television did not predict attention problems	The use of television and video games are not significant predictors of childhood care problems
Engelhardt et al. (96)	Media Use and Sleep Among Boys With Autism Spectrum Disorder, ADHD, or Typical Development	C = United States. *N* = 128 males (8–17 years old). Three groups ASD (n = 49) vs. ADHD (*n* = 38) vs. control group (*n* = 41). T = Descriptive observational study. Task: To study the relationship between the pathology and the use of media and the effectiveness of sleep	A negative correlation was found between the presence of electronic devices in the bedroom and the hours of sleep (*p* < 0.001), number of hours of video games and hours of sleep (*p* > 0.0001)	The presence of electronic devices can cause sleep problems
Ferguson and Olson (97)	Video Game Violence Use Among “Vulnerable” Populations: The Impact of Violent Games on Delinquency and Bullying Among Children with Clinically Elevated Depression or Attention Deficit Symptoms	C = United States. *N* = 377 (12–93 years old). M/F = 140 males/234 females. T = Descriptive observational study. Task: study of video game use and violence	There was no significant correlation between video game use in children with mental health problems and increased violence (*p* = 0.53)	Children with mental health problems do not appear to be a “vulnerable” population to the violent effects of video games
Kietglaiwansiri and Chonchaiya (103)	Pattern of video game use in children with ADHD and typical development Short running title: Videogaming in children with ADHD.	C = Thailand *N* = 182 (6–19 years old). Two groups: ADHD (*n* = 80) vs. control group (*N* = 102). M/F = 91 males/91 females. T = Descriptive observational study. Task: Study of the behavioral pattern with video games in children with ADHD	Children diagnosed with ADHD had greater compulsive behavior when playing video games (37.5% vs. 11.8% *p* < 0.001)	Children with ADHD have more problems using video games
Becker et al. (98)	Nighttime Media Use in Adolescents with ADHD: Links to Sleep Problems and Internalizing Symptoms	C = United States. N = 81 adolescents with ADHD M/F = 56 males/25 females. T = Descriptive study	Adolescents had an average of 5.31 h of use. 77% of the teens reported sleeping less than 8 h. Increased media use has been associated with decreased sleep and increased anxiety (*p* = 0.01), depression (*p* = 0.04) and generalized anxiety reported by parents (*p* = 0.04)	Night-time media use by adolescents with ADHD can lead to sleep problems and internalizing comorbid symptoms
Ferguson and Wang (99)	Aggressive Video Games Are Not a Risk Factor for Mental Health Problems in Youth: A Longitudinal Study	C = Singapore N = 3038 youth M/F = 72.8% male/27.3% female T = Longitudinal study	No link between aggressivity and video game use	
**Level V: Expert opinion**				
Schmidt and Vandewater (100)	Media and Attention, Cognition, and School Achievement	C = United States. T = Narrative text	Influence of video games, television and internet access on the acquisition of cognitive skills in childhood and adolescence	The content of new technologies is more influential than the technology itself
Valkenburg (101)	The Limited Informativeness of Meta-Analyses of Media Effects	C = Netherlands T = Commentary on meta-analysis.	Inconsistent results on the relationship of video game use with aggression and symptoms of inattention	Need for more studies on the effects of video games

*ASD, autism spectrum disorder; PVP, Problem Video Game Playing Questionnaire; YIAS-K, Young Internet Addiction Scale; CPT, Conners’ Performance Test; ASRS, Adult ADHD Self-Report Scale; PG, pathological gambling; NAA-N, acetyl-aspartate.*

## Discussion

The present systematic review suggests that serious video games may be used as effective, playful therapeutic tools for patients with ADHD. In keeping with Strahler Rivero et al. (36), we also found that the use of serious video games for health focuses mainly on the diagnosis of and training to reduce problems in attention, memory, impulse control, emotional regulation, and time management, among others (16–19, 21, 26, 47, 48, 76, 78, 80). Furthermore, serious video games may favor adherence to treatment (24, 62, 65) and allow for a more personalized efficient neurocognitive design. It has been observed that these patients, while practicing with video games, show greater difficulties related to the inhibition of tasks (102), compulsive behaviors (103), and the need for continuous and immediate reinforcement for good performance (81, 85). Finally, patients diagnosed with ADHD have a higher risk of addiction to new technologies due to hypoactivity of the cortical regions. This low activity level is related to lack of control of impulses, time management, greater sensitivity to sounds, lights, and immediate rewards, among others (104). The literature agrees that it is the “impulsivity” trait that is most related to addiction to video games (105, 106).

The evidence presented in Table 1 shows how serious video games provide benefits to ADHD patients. This benefit goes beyond improvements in core ADHD symptoms, reaching other domains such as emotional regulation, which in turn leads to improvements in school and social performance. Treatments for ADHD are usually costly in terms of time, energy, and economic resources for patients and families. This way, serious video games may serve as a complementary activity which may reduce those costs.

All of the video games presented in Table 2 showed good properties when assessing ADHD symptoms, as well as related psychological deficits. Also, some of the video games (30, 72) show high correlations with objective ADHD tests, suggesting the possibilities for designing video games which perform as valid tests for ADHD factors and symptoms.

Table 3 shows the wide variety of psychological deficits associated with ADHD. The most relevant and prominent are, apart from core ADHD symptoms, deficits in attention and memory, proneness to make risky decisions, and a more pronounced sensibility to both positive and negative reinforcement. As seen above, most serious video games reviewed in the present work were found to improve these deficits in inattention, hyperactivity, impulsivity, and executive functions, among others. Concerning decision making, our review did not find serious video games applied to ADHD samples. Nonetheless, a recent review (107) found that serious simulation games improved decision making on samples of professionals like doctors and nurses. However, a generalization of this effect to ADHD patients should be taken with caution. Last, sensibility to reinforcement should be taken into account, more than treated, when designing video games with rewards (Sújar et al., submitted^4^).

Adherence to treatment is improved, taking special care on a series of features of serious video games (this assertion also applies for commercial video games). A motivating general theme for the game (space, pirates, etc.) can greatly improve adherence, as well as the use of frequent and immediate rewards and novelty in challenges (Sújar et al., submitted) (see text footnote 4). About serious video games features with an effective impact on ADHD improvements, two caveat should be considered. First, serious video games tend to get important inspiration from cognitive treatment tasks already validated (which, in turn, improves validity for serious video games), and second, in general terms, immediate feedback and rewards improve not only adherence but also performance, particularly in ADHD patients (Sújar et al., submitted) (see text footnote 4). Nevertheless, scientific literature on the specific topic of video game components and its influence on clinical improvements is scarce and falls beyond the scope of the present review, so our discussion on the matter should be taken with caution.

Finally, we must pay attention to the problems that derive from an excessive use of new technologies in the general population, and in particular in ADHD. Several investigations show how patients with ADHD are more prone to addiction to video games (7, 25, 108). As exposed above, video games, and particularly their abuse, may have a number of negative consequences (109). Nonetheless, Ruiz-Manrique et al. (53) found that therapy with serious video games may decrease abuse of video games. Concerta (110) and psychological therapy and psychoeducation (111) seem to have a similar effect.

Males seem to be more affected by video game addiction (108, 112, 113). This is consistent with the greater prevalence of ADHD in males than females (4). Also, ADHD symptomatology differs between males and females (114, 115). Evidence suggests that males tend to start playing video games earlier, whereas females’ progression to video game addiction tends to be faster (116). In males, time management seems to modulate the relationship between ADHD symptoms and video game addiction, while in females, ADHD modulates the relationship between time management and video game addiction.

In this systematic review we wanted to provide the scientific community with a holistic view of video games and attention-deficit hyperactivity disorder with the main objective of creating new cognitive rehabilitation tools based on video games that are more efficient and motivating for patients. The scope of this review is broader than other recent reviews, covering the appearance of ADHD symptoms and related psychological deficits, as well as their assessment and treatment through serious video games and the benefits of ADHD therapy though video games.

Despite the fact that favorable evidence has grown exponentially in recent years, there are several limitations in published studies such as the lack of replication of studies, the low systematization of research, and low master size, among others.

## Conclusion

Serious video games are a tool for the diagnosis and treatment of ADHD symptoms and other related issues. A therapist should maintain a comprehensive view of video games, including both their problematic use (IGD) but also their potential use as either diagnostic or therapeutic tools. However, the present conclusion is reached after reviewing predominantly male samples, so generalizations to female patients should be made carefully. In the creation and design of a therapeutic video game for patients with ADHD, some relevant aspects should be taken into account: it is necessarily a diagnostic evaluation in order to offer personalized training in those areas and sub-areas more severely affected. The training must be constant over time, the difficulty level must be adjusted to the patient’s competence and in which his progression becomes visible, receiving a positive reinforcement in the first immediate moment and progressively increasing the delay time. In addition, other factors to consider are time management, inhibitory control, reasoning, competitive nature, and transference to situations of daily life, as well as the virtual world in which the game is developed.

## Data Availability Statement

The original contributions presented in the study are included in the article, further inquiries can be directed to the corresponding author.

## Author Contributions

MR-Y and HB-F conceived the original idea and formulated the problem, applied the inclusion and exlusion criteria, and selected the articles for revision. MR-Y designed the search syntaxes. MR-Y and MB-F performed the searches. MR-Y and HB-F wrote the drafts for the manuscript. CG-T and MB-F critically reviewed the manuscript. All authors reviewed and accepted the final version of the manuscript.

## Conflict of Interest

In the last two years, HB-F has received lecture fees from AB-Biotics, Rovi, Praxis, and Shire. He has been paid by Praxis for the design and writing of an article. He was the recipient of a FIPSE Grant (www.fipse.es) in 2018. CG-T and HB-F were employed by Consulting Asistencial Sociosanitario SL. The remaining authors declare that the research was conducted in the absence of any commercial or financial relationships that could be construed as a potential conflict of interest.

## Publisher’s Note

All claims expressed in this article are solely those of the authors and do not necessarily represent those of their affiliated organizations, or those of the publisher, the editors and the reviewers. Any product that may be evaluated in this article, or claim that may be made by its manufacturer, is not guaranteed or endorsed by the publisher.

## Footnotes

1
http://www.prisma-statement.org

2
https://scholar.google.es/

3
https://www.elsevier.com/__data/promis_misc/Levels_of_Evidence.pdf

4 Sújar A, Martín-Moratinos M, Rodrigo-Yanguas M, Bella-Fernández M, González-Tardón C, Delgado-Gómez D, et al. A general tutorial guide for the development of serious video games to treat attention decifit/hyperactivity disorder (ADHD). *JMIR Serious Games.*

## References

[1] Rehbein F Kliem S Baier D Mössle T Petry NM. Prevalence of internet gaming disorder in German adolescents: diagnostic contribution of the nine DSM-5 criteria in a state-wide representative sample. *Addiction.* (2015) 110:842–51. 10.1111/add.12849 25598040

[2] Andreassen CS Billieux J Griffiths MD Kuss DJ Demetrovics Z Mazzoni E The relationship between addictive use of social media and video games and symptoms of psychiatric disorders: a large-scale cross-sectional study. *Psychol Addict Behav.* (2016) 30:252–62. 10.1037/adb0000160 26999354

[3] Weinstein A Weizman A. Emerging association between addictive gaming and attention-deficit/hyperactivity disorder. *Curr Psychiatry Rep.* (2012) 14:590–7. 10.1007/s11920-012-0311-x 22843540

[4] Polanczyk G De Lima MS Horta BL Biederman J Rohde LA. The worldwide prevalence of ADHD: a systematic review and metaregression analysis. *Am J Psychiatry.* (2007) 164:942–8. 10.1176/ajp.2007.164.6.942 17541055

[5] Zayeni D Raynaud J-P Revet A. Therapeutic and preventive use of video games in child and adolescent psychiatry: a systematic review. *Front Psychiatry.* (2020) 11:36. 10.3389/fpsyt.2020.00036 32116851 PMC7016332

[6] Bioulac S Arfi L Bouvard MP. Attention deficit/hyperactivity disorder and video games: a comparative study of hyperactive and control children. *Eur Psychiatry.* (2008) 23:134–41. 10.1016/j.eurpsy.2007.11.002 18206354

[7] Menendez-García A Jiménez-Arroyo A Rodrigo-Yanguas M Marin-Vila M Sánchez-Sánchez F Roman-Riechmann E Internet, video game and mobile phone addiction in children and adolescents diagnosed with ADHD: a case-control study. *Adicciones.* (2020):1469. 10.20882/adicciones.1469 [Epub ahead of print]. 33338245

[8] Delgado-Gómez D Sújar A Ardoy-Cuadros J Bejarano-Gómez A Aguado D Miguélez-Fernández C Objective assessment of attention-deficit hyperactivity disorder (ADHD) using an infinite runner-based computer game: a pilot study. *Brain Sci.* (2020) 10:716. 10.3390/brainsci10100716 33050130 PMC7599622

[9] Wilkinson N Ang RP Goh DH. Online video game therapy for mental health concerns: a review. *Int J Soc Psychiatry.* (2008) 54:370–82. 10.1177/0020764008091659 18720897

[10] García-Ríos CA García-Ríos VE. Videojuegos para niños con trastorno por déficit de atención e hiperactividad. *Dominio de las Ciencias.* (2020) 6:706–17.

[11] Lau HM Smit JH Fleming TM Riper H. Serious games for mental health: are they accessible, feasible, and effective? A systematic review and meta-analysis. *Front Psychiatry.* (2017) 7:209. 10.3389/fpsyt.2016.00209 28149281 PMC5241302

[12] Lim CG Lim-Ashworth NS Fung DS. Updates in technology-based interventions for attention deficit hyperactivity disorder. *Curr Opin Psychiatry.* (2020) 33:577. 10.1097/YCO.0000000000000643 32858596 PMC7575028

[13] Pandian GSB Jain A Raza Q Sahu KK. Digital health interventions (DHI) for the treatment of attention deficit hyperactivity disorder (ADHD) in children-a comparative review of literature among various treatment and DHI. *Psychiatry Res.* (2021) 297:113742. 10.1016/j.psychres.2021.113742 33515870

[14] Vajawat B Varshney P Banerjee D. digital gaming interventions in psychiatry: evidence, applications and challenges. *Psychiatry Res.* (2020) 295:113585. 10.1016/j.psychres.2020.113585 33303223

[15] Villani D Carissoli C Triberti S Marchetti A Gilli G Riva G. Video games for emotion regulation: a systematic review. *Games Health J.* (2018) 7:85–99. 10.1089/g4h.2017.0108 29424555

[16] Prins PJM Ten Brink E Dovis S Ponsioen A Geurts HM De Vries M “braingame brian”: toward an executive function training program with game elements for children with ADHD and cognitive control problems. *Games Health J.* (2013) 2:44–9. 10.1089/g4h.2013.0004 26196554

[17] van der Oord S Ponsionen AJGB Geurts HM Ten Brink EL Prins PJMA. Pilot study of the efficacy of a computerized executive functioning remediation training with game elements for children with ADHD in an outpatient setting:outcome on parent- and teacher-rated executive functioning and ADHD behavior. *J Atten Disord.* (2014) 18:699–712. 10.1177/1087054712453167 22879577

[18] Bul KCM Franken IHA Van der Oord S Kato PM Danckaerts M Vreeke LJ Development and user satisfaction of “plan-it commander,” a serious game for children with ADHD. *Games Health J.* (2015) 4:502–12. 10.1089/g4h.2015.0021 26325247

[19] Healey DM Halperin JM. Enhancing neurobehavioral gains with the aid of games and exercise (ENGAGE): initial open trial of a novel early intervention fostering the development of preschoolers’ self-regulation. *Child Neuropsychol.* (2015) 21:465–80. 10.1080/09297049.2014.906567 24735230

[20] Weerdmeester J Cima M Granic I Hashemian Y Gotsis M. A feasibility study on the effectiveness of a full-body videogame intervention for decreasing attention deficit hyperactivity disorder symptoms. *Games Health J.* (2016) 5:258–69. 10.1089/g4h.2015.0103 27304677

[21] Sánchez-López M Pardo-Guijarro MJ Del Campo DGD Silva P Martínez-Andrés M Gulías-González R Physical activity intervention (Movi-Kids) on improving academic achievement and adiposity in preschoolers with or without attention deficit hyperactivity disorder: study protocol for a randomized controlled trial. *Trials.* (2015) 16:456. 10.1186/s13063-015-0992-7 26458986 PMC4603580

[22] Rivera-Flores GW Vera-Alvarez AE. Intervención computarizada para mejorar la atención sostenida en un niño con TDAH. *Rev Psicol Clín con Niños Adolesc.* (2019) 6:16–22.

[23] Rajabi S Pakize A Moradi N. Effect of combined neurofeedback and game-based cognitive training on the treatment of ADHD: a randomized controlled study. *Appl Neuropsychol Child.* (2020) 9:193–205. 10.1080/21622965.2018.1556101 30734583

[24] Flynn RM Colón-Acosta N Zhou J Bower J. A game-based repeated assessment for cognitive monitoring: initial usability and adherence study in a summer camp setting. *J Autism Dev Disord.* (2019) 49:2003–14. 10.1007/s10803-019-03881-w 30656527

[25] Peñuelas-Calvo I Jiang-Lin LK Girela-Serrano B Delgado-Gomez D Navarro-Jimenez R Baca-Garcia E Video games for the assessment and treatment of attention-deficit/hyperactivity disorder: a systematic review. *Eur Child Adolesc Psychiatry.* (2020) 31:5–20. 10.1007/s00787-020-01557-w 32424511

[26] Kerns KA Price KJ. An investigation of prospective memory in children with ADHD. *Child Neuropsychol.* (2001) 7:162–71. 10.1076/chin.7.3.162.8744 12187473

[27] Cogmed. *CogMed Users.* Stockholm: Cogmed (2012).

[28] García-Redondo P García T Areces D Núñez JC Rodríguez C. Serious games and their effect improving attention in students with learning disabilities. *Int J environmental Res Public Health.* (2019) 16:2480. 10.3390/ijerph16142480 31336804 PMC6679141

[29] Berger I Goldzweig G. Objective measures of attention-deficit/hyperactivity disorder: a pilot study. *IMAJ Israel Med Assoc J.* (2010) 12:531. 21287795

[30] Delgado-Gomez D Peñuelas-Calvo I Masó-Besga AE Vallejo-Oñate S Tello IB Duarte EA Microsoft kinect-based continuous performance test: an objective attention deficit hyperactivity disorder assessment. *J Med Internet Res.* (2017) 19:e79. 10.2196/jmir.6985 28320691 PMC5379015

[31] Pollak Y Weiss PL Rizzo AA Weizer M Shriki L Shalev RS The utility of a continuous performance test embedded in virtual reality in measuring ADHD-related deficits. *J Dev Behav Pediatr.* (2009) 30:2–6. 10.1097/DBP.0b013e3181969b22 19194324

[32] Nolin P Stipanicic A Henry M Lachapelle Y Lussier-Desrochers D Allain P. ClinicaVR: classroom-CPT: a virtual reality tool for assessing attention and inhibition in children and adolescents. *Comput Hum Behav.* (2016) 59:327–33. 10.1016/j.chb.2016.02.023

[33] Díaz-Orueta U Garcia-López C Crespo-Eguílaz N Sánchez-Carpintero R Climent G Narbona J. AULA virtual reality test as an attention measure: convergent validity with conners’ continuous performance test. *Child Neuropsychol.* (2014) 20:328–42. 10.1080/09297049.2013.792332 23638628

[34] Kollins SH DeLoss DJ Cañadas E Lutz J Findling RL Keefe RS A novel digital intervention for actively reducing severity of paediatric ADHD (STARS-ADHD): a randomised controlled trial. *Lancet Digital Health.* (2020) 2(4):e168-e78. 33334505 10.1016/S2589-7500(20)30017-0

[35] Rodrigo-Yanguas M Martín-Moratinos M Menéndez-García A González-Tardón C Sánchez-Sánchez F Royuela A A virtual reality serious videogame versus online chess augmentation in patients with ADHD: a randomized clinical trial. *Games Health J.* (2021) 10:283–92. 10.1089/g4h.2021.0073 34370610

[36] Strahler Rivero T Herrera Nuñez LM Pires EU Amodeo Bueno OF. ADHD rehabilitation through video gaming: a systematic review using PriSMA guidelines of the current findings and the associated risk of bias. *Front Psychiatry.* (2016) 6:151. 10.3389/fpsyt.2015.00151 26557098 PMC4614280

[37] Kokol P Vošner HB Završnik J Vermeulen J Shohieb S Peinemann F. Serious game-based intervention for children with developmental disabilities. *Curr Pediatr Rev.* (2020) 16:26–32. 10.2174/1573396315666190808115238 31393252

[38] Ali A Puthusserypady S. A 3D learning playground for potential attention training in ADHD: a brain computer interface approach. In: *Proceedings of the 2015 37th Annual International Conference of the IEEE Engineering in Medicine and Biology Society (EMBC).* (Piscataway, NJ: IEEE) (2015). 10.1109/EMBC.2015.7318302 26736202

[39] Bikic A Christensen TØ Leckman JF Bilenberg N Dalsgaard S. A double-blind randomized pilot trial comparing computerized cognitive exercises to Tetris in adolescents with attention-deficit/hyperactivity disorder. *Nord J Psychiatry.* (2017) 71:455–64. 10.1080/08039488.2017.1328070 28598701

[40] Lim CG Lee TS Guan C Fung DSS Zhao Y Teng SSW A brain-computer interface based attention training program for treating attention deficit hyperactivity disorder. *PLoS One.* (2012) 7:e46692. 10.1371/journal.pone.0046692 23115630 PMC3480363

[41] Qian X Loo BRY Castellanos FX Liu S Koh HL Poh XWW Brain-computer-interface-based intervention re-normalizes brain functional network topology in children with attention deficit/hyperactivity disorder. *Transl Psychiatry.* (2018) 8:1–11. 10.1038/s41398-018-0213-8 30097579 PMC6086861

[42] Rohani DA Sorensen HB Puthusserypady S. Brain-computer interface using P300 and virtual reality: a gaming approach for treating ADHD. In: *Proceedings of the 2014 36th Annual International Conference of the IEEE Engineering in Medicine and Biology Society.* (Piscataway, NJ: IEEE) (2014). 10.1109/EMBC.2014.6944403 25570771

[43] Shalev L Tsal Y Mevorach C. Computerized progressive attentional training (CPAT) program: effective direct intervention for children with ADHD. *Child Neuropsychol.* (2007) 13:382–8. 10.1080/09297040600770787 17564853

[44] Bul KC Doove LL Franken IH Oord SVD Kato PM Maras A. A serious game for children with attention deficit hyperactivity disorder: who benefits the most? *PLoS One.* (2018) 13:e0193681. 10.1371/journal.pone.0193681 29543891 PMC5854282

[45] Hahn-Markowitz J Berger I Manor I Maeir A. Efficacy of cognitive-functional (Cog-Fun) occupational therapy intervention among children with ADHD: an RCT. *J Atten Disord.* (2020) 24:655–66. 10.1177/1087054716666955 27637735

[46] Shih C-H Wang S-H Wang Y-T. Assisting children with attention deficit hyperactivity disorder to reduce the hyperactive behavior of arbitrary standing in class with a Nintendo Wii remote controller through an active reminder and preferred reward stimulation. *Res Dev Disabil.* (2014) 35:2069–76. 10.1016/j.ridd.2014.05.007 24881005

[47] Dovis S Van der Oord S Wiers RW Prins PJ. Improving executive functioning in children with ADHD: training multiple executive functions within the context of a computer game. a randomized double-blind placebo controlled trial. *PLoS One.* (2015) 10:e0121651. 10.1371/journal.pone.0121651 25844638 PMC4386826

[48] Bul KC Kato PM Van der Oord S Danckaerts M Vreeke LJ Willems A Behavioral outcome effects of serious gaming as an adjunct to treatment for children with attention-deficit/hyperactivity disorder: a randomized controlled trial. *J Med Internet Res.* (2016) 18:e26. 10.2196/jmir.5173 26883052 PMC4773597

[49] Johnstone SJ Roodenrys SJ Johnson K Bonfield R Bennett SJ. Game-based combined cognitive and neurofeedback training using focus pocus reduces symptom severity in children with diagnosed AD/HD and subclinical AD/HD. *Int J Psychophysiol.* (2017) 116:32–44. 10.1016/j.ijpsycho.2017.02.015 28257875

[50] Kermani FK Mohammadi MR Yadegari F Haresabadi F Sadeghi SM. Working memory training in the form of structured games in children with attention deficit hyperactivity disorder. *Iran J Psychiatry.* (2016) 11:224. 28050182 PMC5206324

[51] Moore AL Carpenter DM II Miller TM Ledbetter C. Clinician-delivered cognitive training for children with attention problems: effects on cognition and behavior from the ThinkRx randomized controlled trial. *Neuropsychiatr Dis Treat.* (2018) 14:1671. 10.2147/NDT.S165418 29983567 PMC6027847

[52] Prins PJ Dovis S Ponsioen A Ten Brink E Van Der Oord S. Does computerized working memory training with game elements enhance motivation and training efficacy in children with ADHD? *Cyberpsychol Behav Soc Netw.* (2011) 14:115–22. 10.1089/cyber.2009.0206 20649448

[53] Ruiz-Manrique G Tajima-Pozo K Montañes-Rada F. Case Report:” ADHD Trainer”: the mobile application that enhances cognitive skills in ADHD patients. *F1000Res.* (2014) 3:283. 10.12688/f1000research.5689.2 26962432 PMC4765719

[54] Smith SD Vitulano LA Katsovich L Li S Moore C Li F A randomized controlled trial of an integrated brain, body, and social intervention for children with ADHD. *J Atten Disord.* (2020) 24:780–94. 10.1177/1087054716647490 27178060 PMC5107355

[55] Shaffer RJ Jacokes LE Cassily JF Greenspan SI Tuchman RF Stemmer PJ. Effect of Interactive metronome^®^ training on children with ADHD. *Am J Occup Ther.* (2001) 55:155–62. 10.5014/ajot.55.2.155 11761130

[56] Wroñska N Garcia-Zapirain B Mendez-Zorrilla A. An iPad-based tool for improving the skills of children with attention deficit disorder. *Int J Environ Res Public Health.* (2015) 12:6261–80. 10.3390/ijerph120606261 26042366 PMC4483700

[57] Kaduson HG Finnerty K. Self-control game interventions for attention-deficit hyperactivity disorder. *Int J Play Ther.* (1995) 4:15. 10.1037/h0089359

[58] Bruce C Unsworth C Dillon M Tay R Falkmer T Bird P Hazard perception skills of young drivers with attention deficit hyperactivity disorder (ADHD) can be improved with computer based driver training: an exploratory randomised controlled trial. *Accid Anal Prev.* (2017) 109:70–7. 10.1016/j.aap.2017.10.002 29040873

[59] Larose S Gagnon S Ferland C Pépin M. Psychology of computers: XIV. Cognitive rehabilitation through computer games. *Percept Mot Skills.* (1989) 69:851–8. 10.2466/pms.1989.69.3.851 2608401

[60] Bikic A Leckman JF Christensen TØ Bilenberg N Dalsgaard S. Attention and executive functions computer training for attention-deficit/hyperactivity disorder (ADHD): results from a randomized, controlled trial. *Eur Child Adolesc Psychiatry.* (2018) 27:1563–74. 10.1007/s00787-018-1151-y 29644473

[61] Benzing V Schmidt M. The effect of exergaming on executive functions in children with ADHD: a randomized clinical trial. *Scand J Med Sci Sports.* (2019) 29:1243–53. 31050851 10.1111/sms.13446

[62] Dovis S Maric M Prins PJ Van der Oord S. Does executive function capacity moderate the outcome of executive function training in children with ADHD? *Atten Defic Hyperact Disord.* (2019) 11:445–60. 10.1007/s12402-019-00308-5 31123915

[63] Buitelaar J. Optimising treatment strategies for ADHD in adolescence to minimise ‘lost in transition’ to adulthood. *Epidemiol Psychiatr Sci.* (2017) 26:448–52. 28413998 10.1017/S2045796017000154 PMC6998898

[64] López S Cervantes J-A Cervantes S Molina J Cervantes F. The plausibility of using unmanned aerial vehicles as a serious game for dealing with attention deficit-hyperactivity disorder. *Cogn Syst Res.* (2020) 59:160–70.

[65] Frutos-Pascual M Zapirain BG Zorrilla AM. Adaptive tele-therapies based on serious games for health for people with time-management and organisational problems: preliminary results. *Int J Environ Res Public Health.* (2014) 11:749–72. 24402063 10.3390/ijerph110100749 PMC3924472

[66] Bossenbroek R Wols A Weerdmeester J Lichtwarck-Aschoff A Granic I van Rooij MM. Efficacy of a virtual reality biofeedback game (DEEP) to reduce anxiety and disruptive classroom behavior: single-case study. *JMIR Ment Health.* (2020) 7:e16066. 32207697 10.2196/16066 PMC7139423

[67] Bégel V Di Loreto I Seilles A Dalla Bella S. Music games: potential application and considerations for rhythmic training. *Front Hum Neurosci.* (2017) 11:273. 10.3389/fnhum.2017.00273 28611610 PMC5447290

[68] Savulich G Piercy T Brühl A Fox C Suckling J Rowe JB Focusing the neuroscience and societal implications of cognitive enhancers. *Clin Pharmacol Ther.* (2017) 101:170–2. 27557349 10.1002/cpt.457

[69] Walker JM Bardos AN. Test and product review: Freer, P. (2003). Play attention interactive learning tool. Asheville, NC: Unique Logic and Technology Inc. *J Attent Disord.* (2008) 12:191–3. 10.1177/1087054708316243 18448860

[70] Benzing V Schmidt M. Cognitively and physically demanding exergaming to improve executive functions of children with attention deficit hyperactivity disorder: a randomised clinical trial. *BMC Pediatrics.* (2017) 17:8. 10.1186/s12887-016-0757-9 28068954 PMC5223426

[71] Goodwin A Salomone S Bolton P Charman T Jones EJ Pickles A Attention training for infants at familial risk of ADHD (INTERSTAARS): study protocol for a randomised controlled trial. *Trials.* (2016) 17:608. 10.1186/s13063-016-1727-0 28031039 PMC5192597

[72] Faraone SV Newcorn JH Antshel KM Adler L Roots K Heller M. The groundskeeper gaming platform as a diagnostic tool for attention-deficit/hyperactivity disorder: sensitivity, specificity, and relation to other measures. *J Child Adolesc Psychopharmacol.* (2016) 26:672–85. 27105181 10.1089/cap.2015.0174 PMC5069710

[73] Heller MD Roots K Srivastava S Schumann J Srivastava J Hale TS. A machine learning-based analysis of game data for attention deficit hyperactivity disorder assessment. *Games Health.* (2013) 2:291–8. 10.1089/g4h.2013.0058 26196929

[74] Mitchell WG Chavez JM Baker SA Guzman BL Azen SP. Reaction time, impulsivity, and attention in hyperactive children and controls: a video game technique. *J Child Neurol.* (1990) 5:195–204. 2398235 10.1177/088307389000500308

[75] Mwamba HM Fourie PR van den Heever D. PANDAS: paediatric attention-deficit/hyperactivity disorder application software. *Appl Sci.* (2019) 9:1645. 10.1109/EMBC.2019.8857357 31946165

[76] Peijnenborgh JC Hurks PP Aldenkamp AP van der Spek ED Rauterberg MG Vles JS A study on the validity of a computer-based game to assess cognitive processes, reward mechanisms, and time perception in children aged 4–8 years. *JMIR Serious Games.* (2016) 4:e15. 27658428 10.2196/games.5997 PMC5054232

[77] Serrano-Barroso A Siugzdaite R Guerrero-Cubero J Molina-Cantero AJ Gomez-Gonzalez IM Lopez JC Detecting attention levels in ADHD children with a video game and the measurement of brain activity with a single-channel BCI headset. Sensors. (2021) 21:3221. 10.3390/s21093221 PMC8124980 34066492

[78] Lawrence V Houghton S Douglas G Durkin K Whiting K Tannock R. Executive function and ADHD: a comparison of children’s performance during neuropsychological testing and real-world activities. *J Attent Disord.* (2004) 7:137–49. 10.1177/108705470400700302 15260171

[79] Pop-Jordanova N Gucev Z. Game-based peripheral biofeedback for stress assessment in children. *Pediatr Int.* (2010) 52:428–31. 19863753 10.1111/j.1442-200X.2009.02978.x

[80] Ohan JL Johnston C. What is the social impact of ADHD in girls? A multi-method assessment. *J Abnorm Child Psychol.* (2007) 35:239–50. 17195951 10.1007/s10802-006-9076-1

[81] Aase H Sagvolden T. Infrequent, but not frequent, reinforcers produce more variable responding and deficient sustained attention in young children with attention-deficit/hyperactivity disorder (ADHD). *J Child Psychol Psychiatry.* (2006) 47:457–71. 16671929 10.1111/j.1469-7610.2005.01468.x

[82] Chan PA Rabinowitz T. A cross-sectional analysis of video games and attention deficit hyperactivity disorder symptoms in adolescents. *Ann Gen Psychiatry.* (2006) 5:16. 10.1186/1744-859X-5-16 17059614 PMC1635698

[83] Tahiroglu AY Celik GG Avci A Seydaoglu G Uzel M Altunbas H. Short-term effects of playing computer games on attention. *J Attent Disord.* (2010) 13:668–76. 10.1177/1087054709347205 19773602

[84] Lawrence V Houghton S Tannock R Douglas G Durkin K Whiting K. ADHD outside the laboratory: boys’ executive function performance on tasks in videogame play and on a visit to the zoo. *J Abnorm Child Psychol.* (2002) 30:447–62. 12403149 10.1023/a:1019812829706

[85] Drechsler R Rizzo P Steinhausen H-C. Decision-making on an explicit risk-taking task in preadolescents with attention-deficit/hyperactivity disorder. *J Neural Transm.* (2008) 115:201–9. 17885724 10.1007/s00702-007-0814-5

[86] Lis S Baer N Franzen N Hagenhoff M Gerlach M Koppe G Social interaction behavior in ADHD in adults in a virtual trust game. *J Attent Disord.* (2016) 20:335–45. 10.1177/1087054713482581 23564736

[87] Robaey P McKenzie S Schachar R Boivin M Bohbot VD. Stop and look! Evidence for a bias towards virtual navigation response strategies in children with ADHD symptoms. *Behav Brain Res.* (2016) 298:48–54. 26310386 10.1016/j.bbr.2015.08.019

[88] Furukawa E Alsop B Sowerby P Jensen S Tripp G. Evidence for increased behavioral control by punishment in children with attention-deficit hyperactivity disorder. *J Child Psychol Psychiatry.* (2017) 58:248–57. 27611786 10.1111/jcpp.12635

[89] Silva AP Frère AF. Virtual environment to quantify the influence of colour stimuli on the performance of tasks requiring attention. *Biomed Eng Online.* (2011) 10:74. 10.1186/1475-925X-10-74 21854630 PMC3201025

[90] Dovis S Van der Oord S Wiers RW Prins PJ. Can motivation normalize working memory and task persistence in children with attention-deficit/hyperactivity disorder? The effects of money and computer-gaming. *J Abnorm Child Psychol.* (2012) 40:669–81. 22187093 10.1007/s10802-011-9601-8 PMC3375007

[91] Michel JA Kerns KA Mateer CA. The effect of reinforcement variables on inhibition in children with ADHD. *Child Neuropsychol.* (2005) 11:295–302. 16036453 10.1080/092970490911270

[92] Bolic Baric V Hellberg K Kjellberg A Hemmingsson H. Internet activities during leisure: a comparison between adolescents with ADHD and adolescents from the general population. *J Attent Disord.* (2018) 22:1131–9. 10.1177/1087054715613436 26610742

[93] Swing EL Gentile DA Anderson CA Walsh DA. Television and video game exposure and the development of attention problems. *Pediatrics.* (2010) 126:214–21. 20603258 10.1542/peds.2009-1508

[94] Pfeifer LI Terra LN dos Santos JLF Stagnitti KE Panúncio-Pinto MP. Play preference of children with ADHD and typically developing children in Brazil: a pilot study. *Aust Occup Ther J.* (2011) 58:419–28. 22111644 10.1111/j.1440-1630.2011.00973.x

[95] Ferguson CJ. The influence of television and video game use on attention and school problems: a multivariate analysis with other risk factors controlled. *J Psychiatr Res.* (2011) 45:808–13. 21144536 10.1016/j.jpsychires.2010.11.010

[96] Engelhardt CR Mazurek MO Sohl K. Media use and sleep among boys with autism spectrum disorder, ADHD, or typical development. *Pediatrics.* (2013) 132:1081–9. 24249825 10.1542/peds.2013-2066

[97] Ferguson CJ Olson CK. Video game violence use among “vulnerable” populations: the impact of violent games on delinquency and bullying among children with clinically elevated depression or attention deficit symptoms. *J Youth Adolesc.* (2014) 43:127–36. 23975351 10.1007/s10964-013-9986-5

[98] Becker SP Lienesch JA. Nighttime media use in adolescents with ADHD: links to sleep problems and internalizing symptoms. *Sleep Med.* (2018) 51:171–8. 30223187 10.1016/j.sleep.2018.06.021 PMC6431533

[99] Ferguson CJ Wang CJ. Aggressive video games are not a risk factor for mental health problems in youth: a longitudinal study. *Cyberpsychol Behav Soc Netw.* (2021) 24:70–3. 33252268 10.1089/cyber.2020.0027

[100] Schmidt ME Vandewater EA. Media and attention, cognition, and school achievement. *Future Child.* (2008) 18:63–85. 10.1353/foc.0.0004 21338006

[101] Valkenburg PM. The limited informativeness of meta-analyses of media effects. *Perspect Psychol Sci.* (2015) 10:680–2. 26386007 10.1177/1745691615592237

[102] Shaw R Grayson A Lewis V. Inhibition, ADHD, and computer games: the inhibitory performance of children with ADHD on computerized tasks and games. *J Attent Disord.* (2005) 8:160–8. 10.1177/1087054705278771 16110046

[103] Kietglaiwansiri T Chonchaiya W. Pattern of video game use in children with attention-deficit-hyperactivity disorder and typical development. *Pediatr Int.* (2018) 60:523–8. 29573063 10.1111/ped.13564

[104] Makris N Biederman J Monuteaux MC Seidman LJ. Towards conceptualizing a neural systems-based anatomy of attention-deficit/hyperactivity disorder. *Dev Neurosci.* (2009) 31:36–49. 19372685 10.1159/000207492 PMC3777416

[105] Romo L Rémond J-J Coeffec A Kotbagi G Plantey S Boz F Gambling and Attention Deficit Hyperactivity Disorders (ADHD) in a population of French students. *J Gambl Stud.* (2015) 31:1261–72. 25466366 10.1007/s10899-014-9515-9

[106] Yen J-Y Liu T-L Wang P-W Chen C-S Yen C-F Ko C-H. Association between Internet gaming disorder and adult attention deficit and hyperactivity disorder and their correlates: impulsivity and hostility. *Addict Behav.* (2017) 64:308–13. 27179391 10.1016/j.addbeh.2016.04.024

[107] Reynaldo C Christian R Hosea H Gunawan AA. Using video games to improve capabilities in decision making and cognitive skill: a literature review. *Procedia Comput Sci.* (2021) 179:211–21.

[108] Mathews CL Morrell HE Molle JE. Video game addiction, ADHD symptomatology, and video game reinforcement. *Am J Drug Alcohol Abuse.* (2019) 45:67–76. 29874473 10.1080/00952990.2018.1472269

[109] Villani VS Olson CK Jellinek MS. Media literacy for clinicians and parents. *Child Adolesc Psychiatr Clin.* (2005) 14:523–53. 10.1016/j.chc.2005.03.001 15936672

[110] Han DH Lee YS Na C Ahn JY Chung US Daniels MA The effect of methylphenidate on internet video game play in children with attention-deficit/hyperactivity disorder. *Compr Psychiatry.* (2009) 50:251–6. 19374970 10.1016/j.comppsych.2008.08.011

[111] Pluhar E Kavanaugh JR Levinson JA Rich M. Problematic interactive media use in teens: comorbidities, assessment, and treatment. *Psychol Res Behav Manage.* (2019) 12:447. 10.2147/PRBM.S208968 PMC6615461 31308769

[112] Masi L Abadie P Herba C Emond M Gingras M-P Amor LB. Video games in ADHD and non-ADHD children: modalities of use and association with ADHD symptoms. *Front Pediatr.* (2021) 9:632272. 10.3389/fped.2021.632272 33777866 PMC7994285

[113] Tolchinsky A Jefferson SD. Problematic video game play in a college sample and its relationship to time management skills and attention-deficit/hyperactivity disorder symptomology. *Cyberpsychol Behav Soc Netw.* (2011) 14:489–96. 21288135 10.1089/cyber.2010.0315

[114] Gaub M Carlson CL. Gender differences in ADHD: a meta-analysis and critical review. *J Am Acad Child Adolesc Psychiatry.* (1997) 36:1036–45. 9256583 10.1097/00004583-199708000-00011

[115] Ng QX Ho CYX Chan HW Yong BZJ Yeo W-S. Managing childhood and adolescent attention-deficit/hyperactivity disorder (ADHD) with exercise: a systematic review. *Complement Ther Med.* (2017) 34:123–8. 28917364 10.1016/j.ctim.2017.08.018

[116] Black DW Shaw M Coryell W Crowe R McCormick B Allen J. Age at onset of DSM-IV pathological gambling in a non-treatment sample: early-versus later-onset. *Compr Psychiatry.* (2015) 60:40–6. 25956751 10.1016/j.comppsych.2015.04.007 PMC4459896

